# Bio-Inspired Deep Learning for Parkinson’s Disease Detection: A Comparative Study Based on Vocal Biomarkers and Archimedean Spiral Analysis

**DOI:** 10.3390/biomimetics11060369

**Published:** 2026-05-27

**Authors:** Ovidiu-Petru Stan, Marius Misaros, Liviu-Cristian Miclea

**Affiliations:** Faculty of Automation and Computer Science, Technical University of Cluj-Napoca, 400114 Cluj-Napoca, Romania; ovidiu.stan@aut.utcluj.ro (O.-P.S.); marius.misaros@aut.utcluj.ro (M.M.)

**Keywords:** Parkinson’s disease, bio-inspired computing, machine learning, deep learning, convolutional neural network, vocal biomarkers, Archimedean spiral, biomimetics, early diagnosis

## Abstract

Parkinson’s disease (PD) is the second most prevalent neurodegenerative disorder worldwide, and its early diagnosis remains a major challenge due to reliance on subjective clinical assessments. This study proposes a bio-inspired computational framework for automatic PD detection that draws explicit architectural inspiration from two biological systems: the hierarchical tonotopic organization of the human auditory cortex, which motivates the design of a 1D Convolutional Neural Network (CNN) for vocal biomarker analysis, and the basal ganglia–cerebellar motor control circuit, which motivates the selection and design of features extracted from Archimedean spiral drawing tasks. Unlike previous studies that apply standard machine learning techniques without grounding architectural choices in biological mechanisms, the proposed framework establishes a direct mapping between neural processing pathways and model design decisions. A Support Vector Machine (SVM) classifier evaluated on the Kaggle vocal dataset achieved 87% test accuracy with no overfitting, outperforming AdaBoost, Random Forest, KNN, XGBoost, and Decision Trees in terms of generalization. The 1D CNN applied to UCI spiral drawing data achieved 85% test accuracy, with overfitting behavior addressed through architectural regularization strategies including early stopping. A conceptual multimodal fusion architecture integrating both modalities is proposed as a direction for future experimental validation; it was not implemented or experimentally validated within the present study. The primary novelty of the framework resides in this explicit biomimetic grounding, which distinguishes it from existing performance-driven approaches. Results confirm that biologically grounded computational models constitute promising objective decision-support tools for early PD diagnosis.

## 1. Introduction

Parkinson’s disease (PD) is a chronic and progressive neurological disorder characterized by the degeneration of dopaminergic neurons in the substantia nigra, leading to dopamine depletion. The disease is associated with motor symptoms such as tremor, rigidity, and bradykinesia, significantly affecting patients’ quality of life. Although it is more common in older individuals, approximately one in ten cases is diagnosed before the age of 50 [1].

From a clinical perspective, Parkinson’s disease is associated with cardinal motor symptoms, including bradykinesia, resting tremor, muscular rigidity, and postural instability, as well as non-motor manifestations such as cognitive impairment, autonomic dysfunction, and sensory disturbances. Notably, certain non-motor symptoms may precede the onset of overt motor manifestations by several years, indicating that pathological processes are active well before a clinical diagnosis can be established. Disease severity and progression are assessed using standardized instruments, such as the MDS-UPDRS scale [2].

Despite advances in the field of neurology, early diagnosis of Parkinson’s disease remains a major challenge. Conventional methods rely predominantly on subjective clinical assessments, which limit the ability to identify the disease in its early stages. In this context, data-driven and computational approaches offer the opportunity to develop objective, reproducible, and scalable systems to support clinical diagnosis [3].

From a biomimetic perspective, the present study draws explicit and architecturally grounded inspiration from two distinct biological systems implicated in Parkinson’s disease pathophysiology. The first source of inspiration is the hierarchical tonotopic organization of the human auditory cortex, in which successive neuronal layers process acoustic signals at increasing levels of abstraction—from raw frequency components in the primary auditory cortex (A1) to complex spectrotemporal patterns in higher association areas [4]. This biological processing hierarchy directly motivates the design of the proposed 1D Convolutional Neural Network, in which convolutional filters are sized and stacked to capture vocal tremor frequencies characteristic of PD (approximately 4–12 Hz), mimicking the progressive feature abstraction performed by the auditory pathway. The second source of biological inspiration is the basal ganglia–cerebellar motor control circuit, whose degradation in PD produces the resting tremor, rigidity, and bradykinesia that manifest measurably in fine motor tasks such as Archimedean spiral drawing [5]. Rather than treating the spiral task as a generic drawing exercise, the proposed framework extracts features such as angular velocity profiles, pressure variability, and trajectory regularity, which directly reflect the functional impairment of this biological circuit, establishing a principled biomimetic correspondence between neurological deficit and computational feature design. Together, these two bio-inspired design choices distinguish the proposed framework from approaches that apply standard machine learning architectures without grounding methodological decisions in the underlying biological mechanisms of the disease [3].

Recent research has highlighted the potential of non-invasive biomarkers, particularly the analysis of vocal signals and handwriting dynamics. Speech disorders associated with PD produce measurable changes in acoustic characteristics, which can be automatically analyzed through machine learning techniques [6]. Similarly, graphic tasks such as Archimedean spiral drawing reflect fine motor control deficits and enable early differentiation between healthy subjects and PD patients [7].

In light of these considerations, the present study investigates the application of machine learning and deep learning techniques for the detection of Parkinson’s disease, utilizing vocal biomarkers and spiral drawing analysis. The aim of the study is to develop objective and accessible computational methods capable of supporting early diagnosis and clinical decision-making.

The novelty of the present work lies in the explicit biomimetic grounding of both the CNN architecture and the spiral drawing feature selection, establishing a direct and interpretable correspondence between PD pathophysiology and computational design, a contribution absent from existing purely performance-driven approaches.

The main contributions of this work are: (1) a 1D CNN architecture whose filter dimensions and layer depth are explicitly derived from the tonotopic organization of the human auditory cortex, establishing a neurologically grounded correspondence between biological auditory processing and computational feature extraction for PD vocal biomarker analysis; (2) a spiral drawing feature extraction framework whose kinematic descriptors are explicitly motivated by the functional impairment of the basal ganglia-cerebellar motor control circuit in PD, rather than selected empirically; (3) a systematic comparative evaluation of six machine learning classifiers on the Kaggle vocal dataset, including 5-fold cross-validation, ROC/AUC analysis, confidence intervals, and McNemar statistical significance testing; and (4) a conceptual multimodal fusion architecture integrating both modalities, proposed as a methodologically grounded direction for future experimental validation.

The remainder of this paper is organized as follows. Section 2 reviews related work. Section 3 describes the proposed biomimetic framework and methodology. Section 4 and Section 5 present the experimental results for the vocal and spiral drawing modalities, respectively. Section 6 discusses conclusions and future research directions.

## 2. Current State of Research

### 2.1. Machine Learning and Deep Learning in Healthcare

Recent advances in Machine Learning (ML) and Deep Learning (DL) have significantly influenced the development of intelligent medical systems, enabling improvements in diagnostic processes, monitoring, and clinical decision support. An important research direction is represented by the Internet of Medical Things (IoMT), which integrates interconnected medical devices and sensors with cloud-based infrastructures to enable large-scale collection and processing of medical data [8]. The scalability and availability offered by cloud-integrated IoMT systems have facilitated the deployment of ML and DL models for continuous monitoring and early disease detection [9].

A wide range of ML and DL algorithms has been investigated in medical applications, including models based on decision trees (C4.5), linear and logistic regression, the K-Nearest Neighbors (KNN) algorithm, Convolutional Neural Networks (CNN), and Deep Belief Networks (DBN) [10]. Model selection must be adapted to the characteristics of the available data, as deep learning architectures require large data volumes to manifest their full potential, while classical ML methods may prove more effective on small-scale datasets [11,12,13].

Disease prediction using ML has been extensively studied for cardiovascular conditions, where algorithms such as Random Forest, logistic regression, and the Naïve Bayes classifier have demonstrated high accuracy on standard datasets, including those from the UCI repository [14,15]. Similar trends have been observed in the diagnosis of pulmonary diseases, including pneumonia, where CNN models applied to chest X-ray images have achieved competitive results, particularly when trained on large data volumes [16,17,18].

### 2.2. Machine Learning and Deep Learning for Parkinson’s Disease Detection

Beyond general medical applications, ML and DL techniques have been increasingly adopted in the analysis of neurological disorders, with Parkinson’s disease (PD) representing a major subject of interest [19]. Smartphone-based frameworks exploiting touchscreen interactions and accelerometer data have demonstrated promising results in the unobtrusive detection of incipient motor deficits associated with PD [20]. These systems have achieved high sensitivity and specificity values, supporting their potential for large-scale screening under real-world conditions [21].

Recent advances in deep learning architectures have led to the development of specialized models for the analysis of human gait kinematics based on video data. In this context, Graph Convolutional Networks (GCN) have been adapted for processing skeletal representations extracted from video sequences, enabling the capture of spatiotemporal dependencies between joints [22]. Asymmetric dual-stream GCN architectural variants have demonstrated the ability to simultaneously model local and global motion features, achieving classification accuracies exceeding 84% on the PD-Walk reference dataset [23]. These approaches demonstrate the feasibility of real-time PD detection using non-invasive vision-based systems [24].

**Figure 1:**
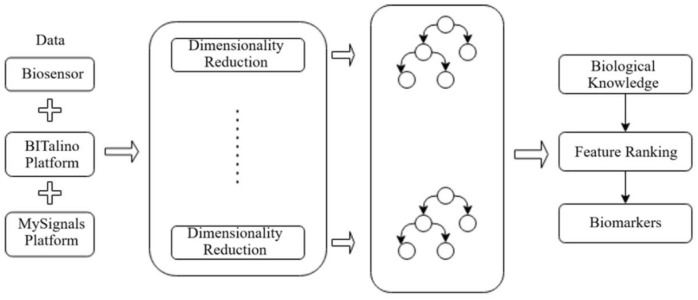
Framework for early-stage Parkinson’s disease detection using ensemble learning methods.

Wearable sensor-based systems have also been investigated for the recognition and severity assessment of Parkinsonian tremor. Accelerometer-based methods combined with ML classifiers have achieved accuracies exceeding 84%, while maintaining low costs and a high degree of patient comfort [25]. Multi-level IoT frameworks integrating biosensors, edge devices, and ML algorithms further support continuous monitoring and early diagnosis of PD, with the described architecture illustrated in Figure 1 [26].

### 2.3. Vocal Biometrics and Parkinson’s Disease Prediction

Voice impairment represents one of the earliest and most frequent non-motor symptoms of Parkinson’s disease, making vocal biomarkers particularly suitable for non-invasive screening applications [27]. Numerous studies have evaluated ML and DL techniques, including SVM, Random Forest, KNN, logistic regression, decision trees, and ensemble-based feature selection methods, for PD detection based on vocal signals [28,29,30,31].

Studies based on MDVP vocal features consistently report Random Forest and SVM as the best-performing classifiers for PD detection, with accuracy values exceeding 90% and high sensitivity scores [32]. Feature selection techniques such as PCA, Boruta, LASSO, and Recursive Feature Elimination have demonstrated the ability to significantly improve classification performance while simultaneously reducing model complexity [33].

Advanced ensemble methods, such as XGBoost, have shown superior robustness on imbalanced datasets, achieving F1 scores exceeding 0.9 [34]. Overall, these results confirm that vocal biomarkers, integrated into appropriate ML pipelines, constitute a reliable and scalable approach for the early diagnosis of Parkinson’s disease [15].

### 2.4. Geometric Indicators Extracted from the Archimedean Spiral

Writing and drawing tasks, particularly Archimedean spiral drawing, have been extensively investigated as indicators of fine motor deficits in Parkinson’s disease. Dynamic and geometric features extracted from drawing trajectories and handwriting have demonstrated a high discriminative capacity between PD patients and healthy subjects [5,35,36].

Studies utilizing graphics tablets and smart pens have highlighted that angular velocity, pressure variation, movement fluency, and tremor frequency components correlate significantly with PD severity, achieving classification accuracies exceeding 90% [5]. Random Forest classifiers applied to geometric and pressure features extracted from the ParkinsonHW dataset have obtained near-perfect AUC values, underscoring both the interpretability and generalization potential of drawing-based biomarkers.

Distance-based methods comparing the reference spiral with the patient’s drawn trajectory have confirmed the sensitivity of angular metrics for the detection of incipient symptoms [37]. Hybrid DL approaches combining CNN architectures with ensemble classifiers have achieved accuracies exceeding 93%, consolidating the effectiveness of multi-stage classification frameworks [38].

## 3. Materials and Methods

### 3.1. Software Environment and Libraries Used

All experiments were conducted using the Python 3.10 (Python Software Foundation, Wilmington, DE, USA) programming language, owing to its widespread adoption in the field of data science and machine learning, as well as the rich ecosystem of scientific libraries available [39].

The experimental workflow was implemented in Jupyter Notebooks6.5.4 (Project Jupyter, San Francisco, CA, USA) [40] and Google Colaboratory (Google LLC, Mountain View, CA, USA) [41], platforms that facilitate reproducibility, collaboration, and cloud-based execution, including GPU acceleration. Numerical computations and matrix operations were performed using the NumPy 1.24.3 (NumFOCUS, Austin, TX, USA) library [42], while data preprocessing and analysis were carried out using Pandas 2.0.1 (NumFOCUS, Austin, TX, USA) [43]. Machine learning models were implemented with Scikit-learn 1.3.0 (INRIA, Paris, France) [44], and data visualization was performed using Matplotlib 3.7.1 (NumFOCUS, Austin, TX, USA) [45].

For the implementation of deep learning models, TensorFlow 2.12.0 (Google LLC, Mountain View, CA, USA) [46] and Keras 2.12.0 (Google LLC, Mountain View, CA, USA) [47] were employed, enabling rapid development of neural network architectures and providing native GPU execution support, facilitating the efficient training of models on large datasets.

### 3.2. Biomimetic Design Rationale

The architectural and methodological choices underlying the proposed framework are explicitly grounded in the biological mechanisms of sensory and motor processing relevant to Parkinson’s disease, rather than being motivated solely by computational performance considerations.

The first biomimetic design principle concerns the processing of vocal biomarkers. The human auditory cortex processes speech signals through a hierarchical pathway in which the primary auditory cortex (A1) performs frequency-selective filtering organized along a tonotopic gradient, while higher cortical areas extract progressively more abstract spectrotemporal representations. In PD patients, dysphonia manifests as measurable perturbations at vocal tremor frequencies ranging from 4 to 7 Hz, alongside increased aperiodicity and elevated noise-to-harmonics ratio [48]. The proposed 1D CNN architecture mirrors this biological hierarchy; brain-inspired neural architectures have demonstrated that integrating biological processing principles directly into network design leads to improved performance and biological plausibility [49]. The first convolutional layer applies filters sensitive to low-level frequency perturbations in the vocal signal, while successive layers extract increasingly abstract feature representations corresponding to higher-order auditory processing. The kernel size and number of filters were selected to align with the frequency resolution required to capture PD-relevant vocal perturbations, establishing a direct correspondence between biological auditory processing and model design.

The second biomimetic design principle concerns the analysis of fine motor control through spiral drawing. In healthy subjects, smooth and coordinated movement is produced by the integrated action of the basal ganglia, which regulate movement initiation and velocity, and the cerebellum, which ensures trajectory precision and error correction [50]. In PD, the progressive degeneration of dopaminergic neurons in the substantia nigra disrupts basal ganglia function, resulting in resting tremor, reduced movement velocity, and irregular trajectory control, all of which are directly observable in spiral drawing tasks [5]. The features extracted in the proposed framework, including spatial coordinate variability, applied pressure fluctuations, pen tilt angle irregularities, and temporal signal spectrograms, are selected to reflect the specific functional deficits of this biological circuit, rather than being chosen on purely statistical grounds.

Together, these two design principles constitute the biomimetic contribution of the present study: a framework in which model architecture and feature selection are motivated by the biological systems whose dysfunction defines Parkinson’s disease, providing a transparent and neurologically interpretable basis for computational PD detection.

### 3.3. Vocal Dataset (Kaggle)

This dataset comprises a series of biomedical voice measurements collected from 31 subjects, of whom 23 were diagnosed with Parkinson’s disease (PD) [51]. The dataset contains a total of 195 voice recordings [52], with each row corresponding to an individual recording and each column associated with a specific vocal feature.

The primary purpose of the dataset is to perform binary classification between healthy subjects and Parkinson’s disease patients, according to the status column, which takes the value 0 for healthy subjects and 1 for patients diagnosed with PD.

**Figure 2:**
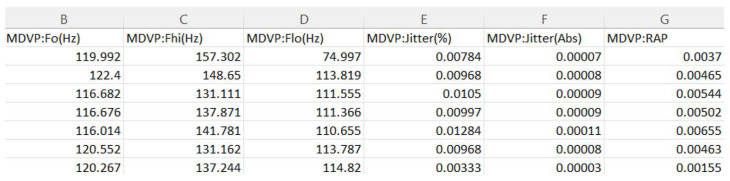
Vocal features from the Kaggle dataset: fundamental frequency and jitter measurements.

**Figure 3:**
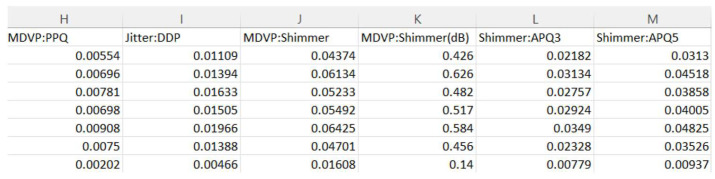
Vocal features from the Kaggle dataset: shimmer measurements.

**Figure 4:**
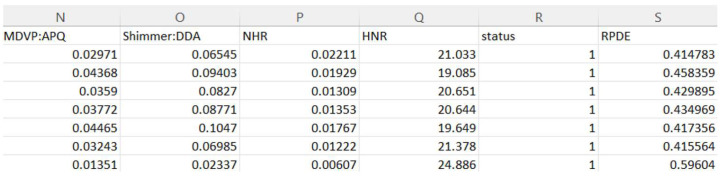
Vocal features from the Kaggle dataset: noise-to-harmonics ratio and nonlinear complexity.

The features included in the dataset are illustrated in Figure 2, Figure 3 and Figure 4 and are described below:

Figure 2:

• Fundamental frequency of the voice: three measurements: MDVP:Fo (Hz), MDVP:Fhi (Hz), MDVP:Flo (Hz);
• Fundamental frequency variation (Jitter): five measurements: MDVP:Jitter (%), MDVP:Jitter (Abs), MDVP:RAP, MDVP:PPQ, Jitter:DDP.

Figure 3:

• Amplitude variation (Shimmer): six measurements: MDVP:Shimmer, MDVP:Shimmer (dB), Shimmer:APQ3, Shimmer:APQ5, MDVP:APQ, Shimmer:DDA;

Figure 4:

• Noise-to-harmonics ratio: two measurements: NHR, HNR;
• Status column: indicates the presence (1) or absence (0) of Parkinson’s disease;
• Nonlinear dynamic complexity: RPDE.

Figure 5:

• Nonlinear dynamic complexity: DFA;
• Nonlinear measurements: spread1, spread2, D2, PPE;

**Figure 5:**
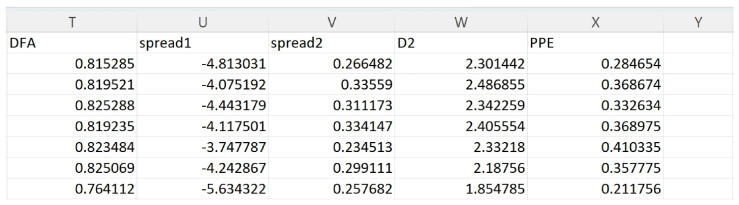
Vocal features from the Kaggle dataset: nonlinear measurements.

### 3.4. Classification Models Used

Two models were implemented for Parkinson’s disease classification: a classical Support Vector Machine (SVM) classifier and a 1D Convolutional Neural Network (1D CNN).

SVM identifies an optimal hyperplane that maximizes the margin of separation between data classes, with the classification decision being determined by the support vectors [53]. Applied to the Kaggle dataset, the model operates in a 22-dimensional space corresponding to the 22 vocal features, performing the separation between vocal recordings of PD patients and those of healthy subjects.

1D CNN was adopted due to its ability to automatically extract relevant features directly from time-domain input data, with reduced computational complexity compared to 2D architectures [54]. The utilized architecture includes convolutional layers for feature extraction, pooling layers for dimensionality reduction, and fully connected layers for final binary classification. From a biomimetic standpoint, the 1D CNN architecture replicates the hierarchical temporal processing performed by the human auditory cortex, where successive neuronal layers extract increasingly abstract representations from raw sensory input, mimicking the biological mechanisms underlying speech perception and motor signal analysis [4].

The hyperparameter configurations adopted for both models are summarized in Table 1. For the SVM classifier, default Scikit-learn parameters were retained, with the RBF kernel selected on the basis of its established suitability for non-linearly separable biomedical feature spaces. For the 1D CNN, architectural parameters including filter sizes, kernel dimensions, learning rate, and regularization strategy were selected based on established practices for small-scale biomedical time-series classification.

In terms of computational complexity, the SVM classifier with RBF kernel has a training complexity of O(n^2^) to O(n^3^) depending on the dataset size, and a prediction complexity of O(n·sv), where sv denotes the number of support vectors. The 1D CNN training complexity is O(e·n·l·f·k), where e is the number of epochs, n the number of samples, l the number of layers, f the number of filters, and k the kernel size. Both models were trained on standard hardware without GPU acceleration, with training times under one minute for the SVM and under five minutes for the CNN. The classification pipelines for both models are described in Algorithm 1 and Algorithm 2, respectively.
**Algorithm 1.** SVM-based vocal biomarker classification pipeline.**Step****Description****Input**Kaggle vocal dataset D (195 samples, 22 features)**1**Load dataset D; separate features X and labels y**2**Apply StandardScaler to X, fitted on training set → X_scaled**3**Split X_scaled into X_train (80%) and X_test (20%), stratified by y**4**Initialize SVM (kernel = RBF, C = 1.0, gamma = scale)**5**Train SVM on (X_train, y_train)**6**Apply stratified 5-fold CV on (X_scaled, y); record accuracy, precision, recall, F1, AUC-ROC**7**Predict y_pred on X_test**8**Compute ROC curve, AUC-ROC, precision-recall curve**Output**Classification results, performance metrics, ROC/PR curves

**Algorithm 2.** 1D CNN-based spiral drawing classification pipeline.
**Step**

**Description**

**Input**
UCI spiral drawing files (control and parkinson folders)
**1**
Load all files; extract columns X, Y, Z, Pressure, GripAngle
**2**
Compute statistical features per column: mean, std, min, max, Q1, Q3
**3**
Assign label 0 (Healthy) for control, 1 (PD) for parkinson
**4**
Apply StandardScaler; reshape to 3D tensor (samples, features, 1)
**5**
Split into X_train (80%) and X_test (20%), stratified by y
**6**
Build 1D CNN: Conv1D(64) → MaxPool → Conv1D(128) → MaxPool → Conv1D(64) → GAP → Dense(64) → Dropout(0.3) → Dense(1, Sigmoid)
**7**
Compile: Adam (lr = 0.001), binary crossentropy loss
**8**
Train with early stopping (patience = 5, max epochs = 50, batch size = 8)
**Output**
Classification results, ROC curve, training history

The broader evolution from classical computer vision techniques to convolution-based models has been comprehensively reviewed in [55], highlighting the superior capacity of deep learning to automatically learn hierarchical representations from data. The application of CNN architectures in medical signal and image analysis has been further consolidated through encoder–decoder designs such as U-Net [56], providing foundational support for the biomimetic CNN-based approach adopted in the present study.

The effectiveness of 1D CNN architectures for vocal biomarker-based PD classification has been further demonstrated on the same Kaggle dataset employed in the present study, confirming the suitability of convolutional feature extraction for this task [57].

### 3.5. Writing Dataset (UCI)

The second dataset used in this study originates from the UCI Machine Learning Repository [58] and includes data collected from 62 patients diagnosed with Parkinson’s disease and 15 healthy subjects, resulting in an imbalanced dataset. The data were obtained using a graphics tablet and aimed to evaluate motor deficits associated with Parkinson’s disease through writing and drawing tasks.

**Figure 6:**
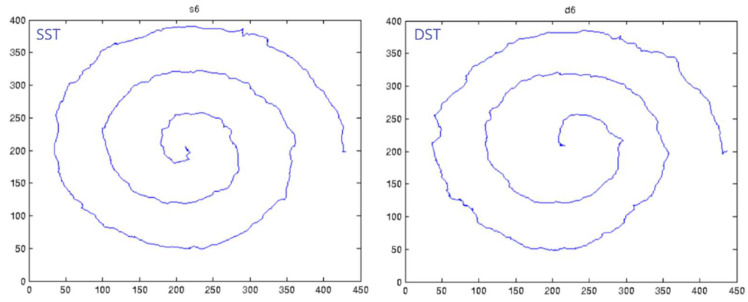
Representative SST and DST spiral drawing samples from the UCI dataset.

For each subject, three types of tests were performed: the Static Spiral Test (SST), the Dynamic Spiral Test (DST), and the Stability Test on a Certain Point (STCP). In the SST, subjects were instructed to trace as accurately as possible an Archimedean spiral displayed on the graphics tablet, a method frequently used in the clinical assessment of motor performance and tremor. The DST involves drawing the spiral under conditions where the reference model is displayed intermittently, allowing the analysis of motor control in the absence of visual guidance, with both tests illustrated in Figure 6.

The STCP consists of holding the digital pen above a fixed point without contact with the tablet surface, and is used to assess hand stability and tremor levels. Data were collected using a graphics tablet that interacts exclusively with digital pens. For each recording, a .txt file is available containing detailed temporal and spatial information about the drawing process. This information is recorded in a three-dimensional coordinate system (XYZ) and includes the X, Y, and Z coordinates of the pen, the pressure applied to the tablet surface, the pen tilt angle, the timestamp, and the test identifier (0—SST, 1—DST, 2—STCP).

**Figure 7:**
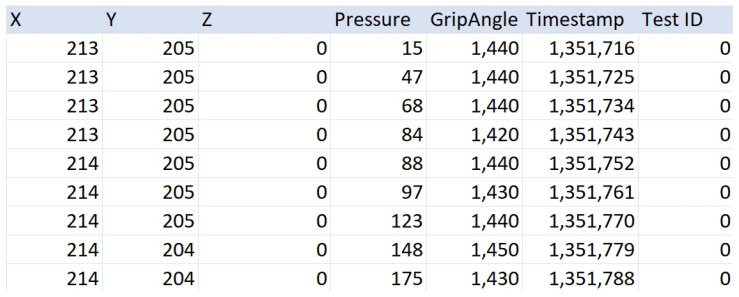
Structure of the .txt recording files from the UCI dataset.

The number of samples per recording varies depending on the duration of the task and can reach several thousand values, as illustrated in Figure 7.

## 4. Methodology for Parkinson’s Disease Detection Based on Vocal Biometrics

### 4.1. Methodology

This chapter presents the implementation methodology of the automatic Parkinson’s disease detection technique based on vocal biomarkers, using the vocal dataset available on the Kaggle platform, described in Section 3. The experimental implementation was carried out in the Google Colaboratory environment, which provides cloud-based execution support and promotes experimental reproducibility.

The proposed method is based on the binary classification of subjects into Parkinson and non-Parkinson classes, with a Support Vector Machine (SVM) model being initially implemented. For comparative purposes, additional machine learning algorithms were evaluated using the same preprocessing and validation strategy.

**Figure 8:**
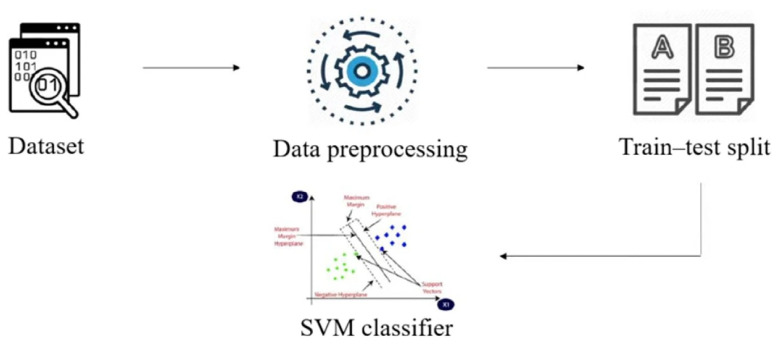
SVM classification process diagram.

The general workflow of the PD detection process is illustrated in Figure 8 and includes the following stages: data loading, descriptive statistical analysis including the evaluation of distributions and basic statistical values of vocal features, preprocessing, splitting into training and testing sets, model training, and performance evaluation.

During the preprocessing stage, the data were analyzed from a structural and statistical perspective, and missing values were handled through mean-based imputation. Numerical features were subsequently standardized using StandardScaler from the Scikit-learn library, fitted exclusively on the training set and applied to both training and validation sets to prevent data leakage. A fixed random seed (random_state = 42) was applied throughout all experiments to ensure full reproducibility of the reported results. The data were split into training and testing sets, allocating 20% of the total recordings for the testing stage, using a reproducible random split controlled by the random_state parameter. The SVM model was trained on the preprocessed data, and its performance was evaluated on a separate test set using standard classification metrics, including accuracy, precision, recall, and F1-score.

It is acknowledged that the Kaggle vocal dataset contains multiple recordings per subject, and that the applied stratified split does not guarantee subject-level separation between training and validation sets. This represents a known limitation that may result in optimistic performance estimates, and subject-independent splitting is identified as a priority methodological improvement for future work.

### 4.2. Validation Strategy and Performance Evaluation

The classification model was evaluated using a stratified data splitting strategy, allocating 80% of recordings for training and 20% for validation, with class proportions preserved across both sets to account for the inherent class imbalance in the dataset. Prior to training, all features were standardized using StandardScaler to ensure uniform scaling and prevent high-magnitude variables from dominating the learning process. Performance was assessed using accuracy scores computed on both the training and test sets, with consistency between the two monitored as an indicator of generalization capacity.

**Figure 9:**
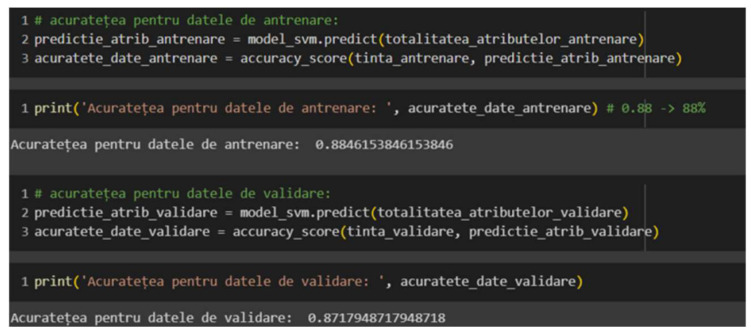
SVM accuracy scores on training and test sets.

For the Support Vector Machine model, training accuracy of 0.88 and test accuracy of 0.87 were obtained, indicating strong generalization capacity and the absence of overfitting—a particularly noteworthy result given the small size of the dataset. The negligible difference between training and test scores suggests that the SVM successfully captured the discriminative patterns in the vocal feature space without adapting excessively to the training data. These results are consistent with findings reported in the literature for SVM-based PD detection on similar vocal datasets [48]. The results are illustrated in Figure 9.

To provide a statistically robust estimate of generalization performance, a stratified 5-fold cross-validation procedure was applied to the SVM model, with class proportions preserved across each fold. The cross-validation results are summarized in Table 2. The mean accuracy of 87.18% (±5.38%) across folds is fully consistent with the single-split result of 87%, confirming that the model’s performance is stable and does not constitute an artifact of a particular train-validation partition. Notably, the mean recall of 99.31% (±1.38%) across folds indicates that the SVM correctly identifies virtually all PD-positive cases, a clinically critical property for a screening tool where false negatives carry significant diagnostic cost.

**Figure 10:**
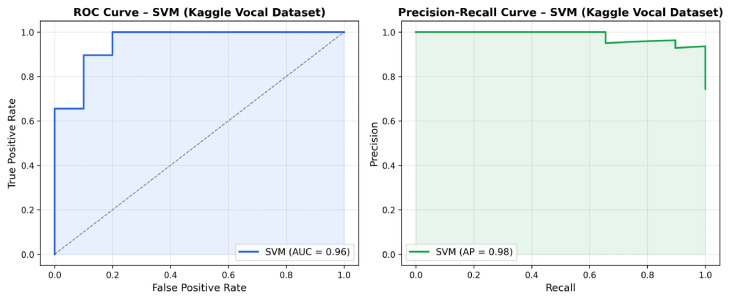
ROC curve and Precision-Recall curve for the SVM classifier on the Kaggle vocal dataset (80/20 stratified split). AUC-ROC = 0.9552. The dashed diagonal line represents the random classifier baseline. The shaded areas indicate the area under the respective curves.

Beyond accuracy, the performance of the SVM classifier was further characterized using the ROC curve and precision-recall curve computed on the 80/20 train-test split, illustrated in Figure 10. The detailed per-class classification metrics are presented in Table 3, with an overall AUC-ROC of 0.9552. The AUC-ROC value confirms strong discriminative capacity across all classification thresholds. The weighted-average precision of 0.93 and recall of 0.92 on the test set confirm balanced performance across both classes, with the classifier achieving perfect precision (1.00) for the healthy class and perfect recall (1.00) for the PD class, indicating that no PD patient was misclassified as healthy, a property of direct clinical relevance given the asymmetric cost of false negatives in diagnostic screening.

Despite the strong performance, the lower recall for the healthy class (0.70) indicates a tendency to classify healthy subjects as PD, a bias partially attributable to class imbalance (147 PD versus 48 healthy recordings) that warrants consideration in clinical screening contexts.

To assess the statistical significance of the performance differences between classifiers, McNemar’s test was applied to compare the SVM model against each competing classifier on the test set. The results are summarized in Table 4. No statistically significant differences were observed (*p* > 0.05 in all comparisons), a result consistent with the limited size of the test set (39 samples) which constrains the statistical power of the test. These results should therefore be interpreted with caution, and significance testing on larger datasets is identified as a direction for future work.

### 4.3. Comparative Analysis of Classifiers

To evaluate the robustness of the proposed method, the performance of the SVM model was compared against other classification algorithms frequently used in the literature for Parkinson’s disease detection. Models such as AdaBoost, Decision Tree, K-Nearest Neighbors (KNN), XGBoost, and Random Forest were implemented and evaluated using the same preprocessing and validation strategy.

The comparative results obtained are summarized in Table 5, where accuracy scores for the training and test sets are presented. It can be observed that the majority of alternative models achieve very high accuracy values on the training data; however, their performance decreases significantly on the test set. This behavior indicates an overfitting phenomenon, particularly characteristic of small and relatively homogeneous datasets.

In contrast, the SVM model demonstrates a superior balance between training and validation performance, confirming its generalization capacity. Although it does not achieve the highest scores on the training set, the stability of its results on unseen data recommends it as the most reliable solution for the problem addressed.

The obtained results can be further contextualized relative to representative studies from the literature that employed the same or similar vocal datasets. Hossain and Amenta [15], working on a speech biomarker dataset of comparable scale, reported validation accuracies of 80.26% for Random Forest and 76.32% for SVM under standard classification pipelines, with the best-performing pipeline (LSVC + AdaBoost combining feature selection with classification) reaching a maximum accuracy of 85.09% and an AUC of 0.90. These results confirm that standard classifiers applied without feature selection optimization on small vocal datasets do not systematically exceed 85% accuracy, and that the 87% test accuracy obtained by the proposed SVM model is competitive within this range. Studies reporting accuracy values above 90% on vocal datasets, such as the 91.83% achieved by Random Forest and the approximately 91.75% achieved by SVM with PCA-based feature selection reported in [32], employed the UCI vocal dataset with a 75/25 train-test split and no cross-validation, conditions that are susceptible to optimistic performance estimates on datasets comprising only 195 recordings from 31 subjects. Similarly, XGBoost-based approaches have been reported to achieve F1-scores of 0.922 with accuracy of 0.880 on imbalanced vocal datasets [34]. In this context, the primary differentiating contribution of the present work is not the absolute classification accuracy, but the explicit biomimetic grounding of the architectural and feature selection choices, which provides a neurologically interpretable basis absent from purely performance-driven approaches, alongside the demonstrated generalization stability evidenced by the negligible difference between training and test accuracy.

## 5. Parkinson’s Disease Detection Technique Based on Spiral Analysis

### 5.1. Methodology and Implementation

The Parkinson’s disease detection technique based on spiral analysis was implemented in the Google Colaboratory environment, using the UCI dataset described in Section 3. The proposed methodology follows three main stages: data preparation, convolutional neural network (CNN) modeling, and model training and optimization.

**Figure 11:**
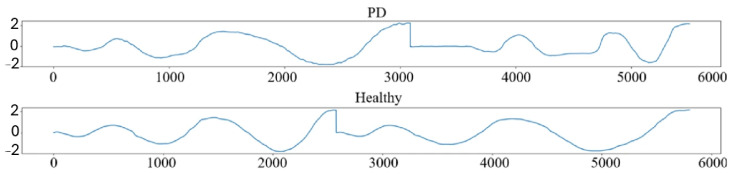
X coordinate variation over time for PD and healthy.

**Figure 12:**
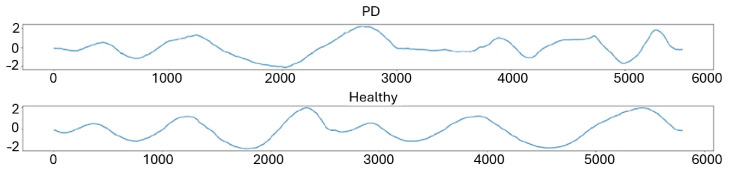
Y coordinate variation over time for PD and healthy.

**Figure 13:**
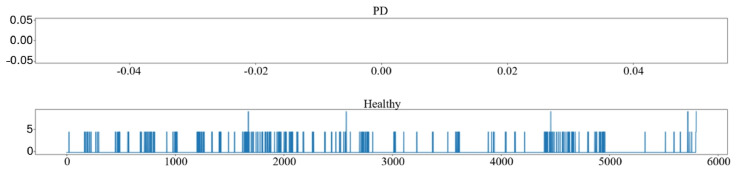
Z coordinate variation over time for PD and healthy.

During the data preparation stage, signals corresponding to spiral drawing tests were analyzed for subjects diagnosed with Parkinson’s disease and control subjects. Relevant attributes were extracted from the raw files, namely the spatial coordinates (X, Y, Z), the applied pressure, and the pen tilt angle. These signals were represented in the time domain, as illustrated in Figure 11, Figure 12 and Figure 13, highlighting significant differences between PD and healthy cases, determined by the impairment of fine motor control.

**Figure 14:**
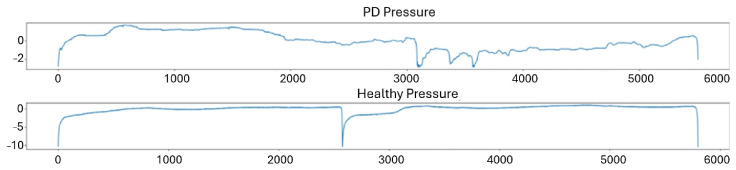
Applied pressure variation over time for PD and healthy.

**Figure 15:**
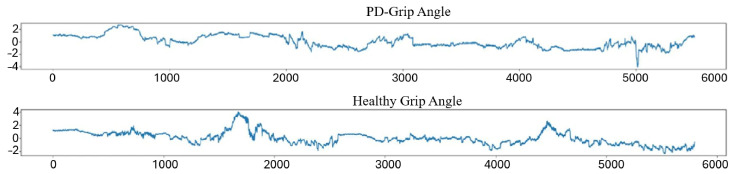
Pen tilt angle variation over time for PD and healthy.

The spatial coordinate signals reveal clear differences between PD and healthy subjects, with PD cases exhibiting irregular oscillatory patterns attributable to tremor and reduced motor control. Similar observations can be noted in the pressure and tilt angle signals presented in Figure 14 and Figure 15, where PD subjects display higher variability and less stable trajectories compared to healthy controls.

**Figure 16:**
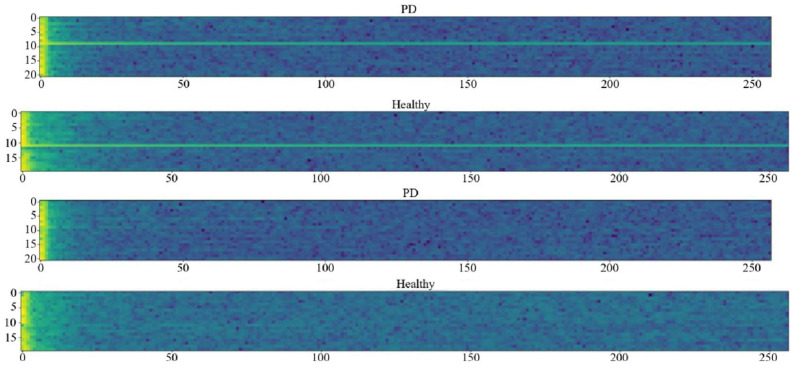
PD/healthy spectrograms for X and Y attributes. The color gradient represents signal intensity, ranging from dark blue (low intensity) to yellow-green (high intensity), expressed in decibels (dB) on the mel scale.

**Figure 17:**
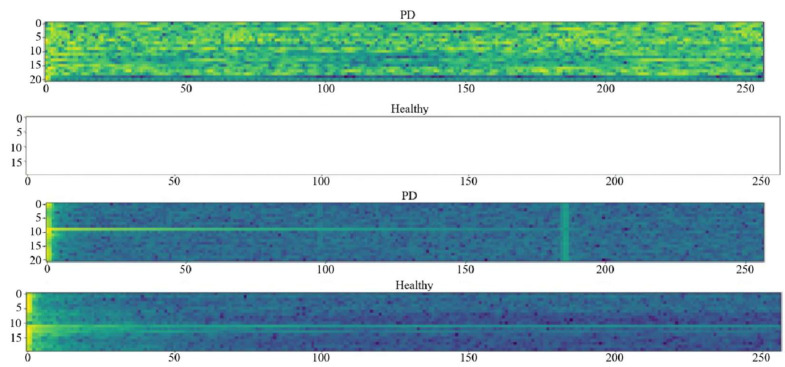
PD/healthy spectrograms for Z and applied pressure. The color gradient represents signal intensity, ranging from dark blue (low intensity) to yellow-green (high intensity), expressed in decibels (dB) on the mel scale.

For a more detailed analysis of the dynamic behavior of the signals, spectrograms were generated for each attribute. Spectrograms provide a time–frequency representation of the signals, highlighting spectral variations associated with tremor and motor instability. These were expressed on the mel scale, which reflects the nonlinear perception of frequency by the human auditory system. The obtained descriptors were used both as one-dimensional features (numerical values) and as two-dimensional features (spectrogram images). Spectrogram generation was performed using the Discrete Fourier Transform, resulting in time–frequency representations illustrated in Figure 16 and Figure 17, in which the horizontal axis corresponds to time and the vertical axis to frequency.

**Figure 18:**
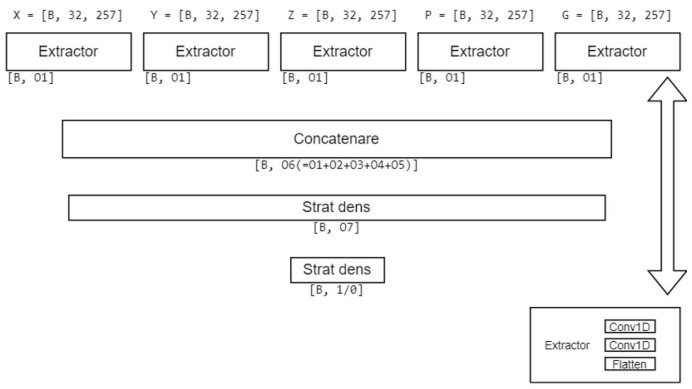
Proposed multi-channel 1D CNN architecture for binary PD/healthy classification.

In the final data preparation stage, data batches were constructed for training and validation, with each batch being composed of signals from multiple PD and healthy subjects. The classification system was modeled using a convolutional neural network, in accordance with the theoretical principles presented in Section 3. The proposed architecture consists of 1D convolutional layers used for the automatic extraction of relevant features, followed by dense layers for classification. Each input associated with the five considered attributes is represented as a three-dimensional tensor, defined by the batch size, time, and frequency dimensions. The outputs of the individual convolutional extractors are concatenated and transmitted to the final dense layer, which produces the binary classification decision (PD/healthy). The general architecture of the model is illustrated in Figure 18.

### 5.2. Validation and Performance Evaluation

**Figure 19:**
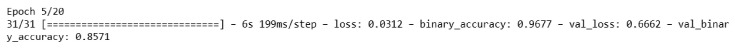
The Highest-Performing Iteration.

The CNN model was trained over 20 epochs, with performance on the test set monitored continuously throughout the training process. The best performance was obtained during the fifth epoch, when a training accuracy of 0.96 and a test accuracy of 0.85 were recorded, as illustrated in Figure 19. Subsequent epochs did not yield further relevant performance improvements, and early stopping was applied to prevent further overfitting.

The observed discrepancy between training accuracy (0.96) and test accuracy (0.85) confirms the presence of overfitting, an expected behavior of deep learning models trained on small biomedical datasets. This was partially mitigated through early stopping with a patience of 5 epochs and a dropout rate of 0.3, which stabilized test performance and prevented further divergence between training and test metrics.

Following the completion of training, the model was applied to new data files, demonstrating the ability to correctly identify individual cases of Parkinson’s disease. In the present study, this behavior is attributable to the limited size of the UCI spiral dataset, which comprises only 77 subjects with a pronounced class imbalance between PD and control subjects. Several architectural mitigation strategies were incorporated into the proposed framework, including early stopping, which contributed to stabilizing the validation performance across epochs.

**Figure 20:**
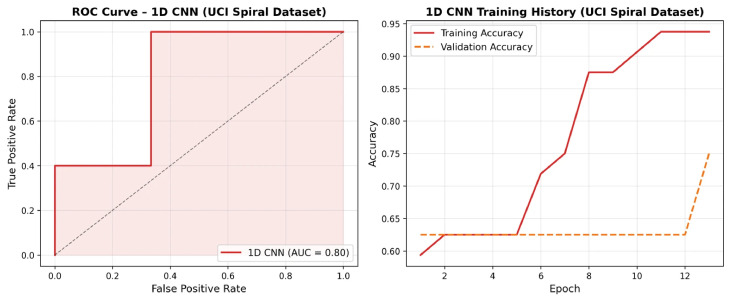
ROC curve (AUC = 0.80) and training/test accuracy history for the 1D CNN classifier on the UCI spiral dataset. The dashed diagonal line represents the random classifier baseline. The shaded area indicates the area under the ROC curve.

The performance of the 1D CNN model was further evaluated using the AUC-ROC metric, with the resulting curve and training history illustrated in Figure 20. An AUC-ROC value of 0.80 was obtained, indicating good discriminative capacity between PD and healthy subjects despite the severely limited dataset size. The model achieved a best test accuracy of 85% at epoch 5, with early stopping preventing overfitting beyond this point.

It is acknowledged that the small number of subjects in the UCI dataset constitutes a significant constraint on the statistical reliability of these results, and that a Leave-One-Subject-Out cross-validation strategy would provide a more robust estimate of generalization performance. This remains a priority direction for future work, as outlined in Section 6.2. These results should be interpreted with caution given the limited dataset size, as class imbalance and small sample size may affect the reliability of the classification metrics.

## 6. Conclusions

### 6.1. Results Obtained

This study proposed a bio-inspired computational framework for automatic PD detection, grounded in two biological systems: the hierarchical tonotopic organization of the auditory cortex, which motivated the 1D CNN design, and the basal ganglia–cerebellar motor control circuit, which motivated the spiral drawing feature selection. Unlike previous studies that apply standard machine learning techniques without grounding architectural choices in biological mechanisms, the proposed framework establishes a principled biomimetic correspondence between neurological deficit and computational design.

The experimental results confirm that both vocal biomarkers and fine motor analysis contain discriminative information for differentiating healthy subjects from PD patients. The SVM classifier achieved 87% test accuracy on the Kaggle vocal dataset, demonstrating strong generalization capacity under limited data conditions and outperforming all compared classifiers in terms of training-test consistency. The 1D CNN applied to UCI spiral drawing data achieved 85% test accuracy, with overfitting behavior addressed through early stopping and acknowledged as a known limitation of deep learning on small biomedical datasets. The comparison of classifiers confirmed that well-regularized classical models can offer superior generalization balance compared to ensemble methods on small-scale biomedical data. The results confirm that biologically grounded models constitute promising decision-support tools for early PD diagnosis. The proposed multimodal fusion architecture remains conceptual and was not experimentally validated within the present study. The individual modality results presented here provide the empirical foundation for its future development and validation.

From a clinical translation perspective, the proposed framework is designed as a decision-support tool rather than a replacement for clinical diagnosis. Both the vocal biomarker analysis and the spiral drawing task can be performed non-invasively using commodity hardware, specifically a standard microphone and a graphics tablet, making the system deployable in primary care or outpatient settings without specialized equipment. However, the datasets used in this study originate from controlled laboratory conditions, and validation on heterogeneous clinical populations acquired across multiple centers remains a necessary step before deployment. The impact of demographic variables such as age, medication status, and disease stage on model performance requires further investigation in future clinical validation studies.

### 6.2. Future Development Directions

External validation on independent datasets acquired from different clinical centers represents a critical direction for future work. The absence of external validation in the present study is acknowledged as a limitation inherent to the availability of public PD datasets, and the reported results should be interpreted within this constraint.

Cross-dataset generalization between the vocal and spiral drawing modalities was not evaluated in the present study, as the two datasets employ fundamentally different feature spaces that are not directly comparable. Future work will address generalization by validating each modality independently on additional publicly available PD datasets.

Calibration analysis of the proposed classifiers, assessing the alignment between predicted probabilities and observed outcomes, was not performed in the present study and represents an additional direction for future work, particularly relevant in the context of clinical deployment where well-calibrated probability estimates are essential for informed decision-making.

Future research directions aim at extending and consolidating the proposed bio-inspired framework along several axes. The primary direction consists of implementing and validating the multimodal fusion architecture proposed in this study, which integrates vocal biomarker analysis and spiral drawing features into a unified classification pipeline. Such a fusion framework would represent a methodologically novel contribution relative to existing single-modality approaches and is expected to improve both classification accuracy and robustness.

A second priority direction concerns the validation strategy. Stratified 5-fold cross-validation has been incorporated for the vocal dataset in the present study, confirming the stability of the SVM results across folds. Future work will extend this to Leave-One-Subject-Out cross-validation for the spiral dataset, providing statistically robust estimates appropriate for the small subject count in the UCI dataset. Additionally, data augmentation techniques and advanced regularization strategies will be explored to mitigate overfitting in the CNN model.

A third direction involves the validation of the proposed models on larger and more diverse datasets sourced from multiple medical centers, in order to improve robustness and generalization capacity across different patient populations. Integration of the developed framework into a user-friendly clinical decision-support application, deployable on desktop and mobile platforms, represents a further direction aimed at increasing the practical impact and accessibility of the solution for medical personnel.

## Figures and Tables

**Table 1:** Hyperparameter configuration for the SVM and 1D CNN models.

Model	Parameter	Value
SVM	Kernel	RBF
SVM	C (regularization)	1.0
SVM	Gamma	scale
SVM	Probability estimates	Enabled
1D CNN	Convolutional layers	3 (filters: 64, 128, 64)
1D CNN	Kernel size	3
1D CNN	Activation	ReLU
1D CNN	Optimizer	Adam (lr = 0.001)
1D CNN	Batch size	8
1D CNN	Max epochs	50
1D CNN	Early stopping patience	5
1D CNN	Dropout rate	0.3

**Table 2:** Stratified 5-fold cross-validation results for the SVM classifier on the Kaggle vocal dataset.

Metric	Mean	Std Dev	95% CI
Accuracy	0.8718	±0.0538	(0.7664, 0.9772)
Precision	0.8610	±0.0494	(0.7642, 0.9578)
Recall	0.9931	±0.0138	(0.9656, 1.000)
F1-Score	0.9217	±0.0315	(0.8600, 0.9834)
AUC-ROC	0.7488	±0.0946	(0.5629, 0.9347)

**Table 3:** Per-class classification metrics for the SVM classifier on the Kaggle test set (80/20 split).

Class	Precision	Recall	F1-Score	Support
Healthy	1.00	0.70	0.82	10
PD	0.91	1.00	0.95	29
Weighted avg	0.93	0.92	0.92	39

**Table 4:** McNemar’s test results comparing SVM against competing classifiers on the Kaggle test set.

Comparison	*p*-Value	Significant (α = 0.05)
SVM vs. AdaBoost	1.0000	No
SVM vs. Random Forest	1.0000	No
SVM vs. KNN	1.0000	No
SVM vs. Decision Tree	0.4531	No

**Table 5:** Comparative accuracy scores of classification models on training and test sets.

Model	Accuracy (Training)	Accuracy (Validation)	Overtrained
SVM	0.88	0.87	NO
AdaBoost	1.00	0.87	YES
Decision Tree	1.00	0.76	YES
KNN	0.95	0.84	YES
XGBoost	1.00	0.82	YES
RF	1.00	0.84	YES

## Data Availability

The vocal dataset is publicly available on Kaggle at: https://www.kaggle.com/datasets/vikasukani/parkinsons-disease-data-set (accessed on 22 October 2025). The spiral drawing dataset is publicly available at the UCI Machine Learning Repository at: https://archive.ics.uci.edu/dataset/395/parkinson+disease+spiral+drawings+using+digitized+graphics+tablet (accessed on 22 December 2025). No new data were created in this study.
